# Biosensing of Alpha-Fetoprotein: A Key Direction toward the Early Detection and Management of Hepatocellular Carcinoma

**DOI:** 10.3390/bios14050235

**Published:** 2024-05-08

**Authors:** Lohit Ramachandran, Farah Abul Rub, Amro Hajja, Ibrahim Alodhaibi, Momo Arai, Mohammed Alfuwais, Tariq Makhzoum, Ahmed Yaqinuddin, Khaled Al-Kattan, Abdullah M. Assiri, Dieter C. Broering, Raja Chinnappan, Tanveer Ahmad Mir, Naresh Kumar Mani

**Affiliations:** 1 Microfluidics, Sensors and Diagnostics (μSenD) Laboratory, Centre for Microfluidics, Biomarkers, Photoceutics and Sensors (μBioPS), Department of Biotechnology, Manipal Institute of Technology, Manipal Academy of Higher Education, Manipal 576104, India; lohit.ramachandran@learner.manipal.edu; 2 College of Medicine, Alfaisal University, Riyadh 11533, Saudi Arabia; fabulrub@alfaisal.edu (F.A.R.); ahajja@alfaisal.edu (A.H.); ialodhaibi@alfaisal.edu (I.A.); marai@alfaisal.edu (M.A.); mohfuwaiz@gmail.com (M.A.); tmakhzoum@alfaisal.edu (T.M.); ayaqinuddin@alfaisal.edu (A.Y.); kkattan@alfaisal.edu (K.A.-K.); assiri@kfshrc.edu.sa (A.M.A.); dbroering@kfshrc.edu.sa (D.C.B.); 3 Lung Health Center Department, Organ Transplant Centre of Excellence, King Faisal Specialist Hospital and Research Centre, Riyadh 11211, Saudi Arabia; 4 Tissue/Organ Bioengineering & BioMEMS Laboratory, Organ Transplant Centre of Excellence (TR&I-Dpt), King Faisal Specialist Hospital and Research Centre, Riyadh 11211, Saudi Arabia

**Keywords:** hepatocellular carcinoma, alpha-fetoprotein, biosensors, early detection, diagnostic tools

## Abstract

Hepatocellular carcinoma (HCC) is currently one of the most prevalent cancers worldwide. Associated risk factors include, but are not limited to, cirrhosis and underlying liver diseases, including chronic hepatitis B or C infections, excessive alcohol consumption, nonalcoholic fatty liver disease (NAFLD), and exposure to chemical carcinogens. It is crucial to detect this disease early on before it metastasizes to adjoining parts of the body, worsening the prognosis. Serum biomarkers have proven to be a more accurate diagnostic tool compared to imaging. Among various markers such as nucleic acids, circulating genetic material, proteins, enzymes, and other metabolites, alpha-fetoprotein (AFP) is a protein marker primarily used to diagnose HCC. However, current methods need a large sample and carry a high cost, among other challenges, which can be improved using biosensing technology. Early and accurate detection of AFP can prevent severe progression of the disease and ensure better management of HCC patients. This review sheds light on HCC development in the human body. Afterward, we outline various types of biosensors (optical, electrochemical, and mass-based), as well as the most relevant studies of biosensing modalities for non-invasive monitoring of AFP. The review also explains these sensing platforms, detection substrates, surface modification agents, and fluorescent probes used to develop such biosensors. Finally, the challenges and future trends in routine clinical analysis are discussed to motivate further developments.

## 1. Introduction

Hepatocellular carcinoma (HCC) is one of the most common cancers in humans with chronic liver diseases and cirrhosis [1]. HCC is the second leading cause of cancer death in men, after lung cancer. Major risk factors of HCC include chronic hepatitis B and hepatitis C viruses and alcoholic and nonalcoholic fatty liver diseases (NAFLD) [2,3,4]. The 5-year survival rate can be increased significantly with early diagnosis and proper treatment. However, in most cases, the disease is not identified before its intermediate or advanced stages, partly due to a clinically asymptomatic early stage [5,6]. Typically, conventional laboratory imaging techniques are used for the diagnosis and prognosis of HCC. Despite their broader acceptance, imaging modalities carry poor sensitivity and specificity [7,8]. Alternatively, serum biomarkers such as proteins, nucleic acids, enzymes, and other metabolites can be used for detection [9,10,11]. Recently, bioengineered organoid models have played a potential tool in liver cancer research. They retain the molecular functions of the native tissues significantly during long-term culture in vitro [12]. Human alpha-fetoprotein (AFP) is one of several HCC marker-associated glycoproteins entangled with both ontogenic and oncogenic growth in men, non-pregnant women, and children [13,14,15]. It is normally found in the embryo yolk sac, and may be aberrantly produced in the liver and digestive tract of humans in various diseases [16]. In healthy people, the serum AFP level typically does not exceed 25 ng/mL [17,18]. However, in more than 75% of patients with HCC, AFP levels increase to 400 ng/mL [19]. Monitoring HCC, on the other hand, is achieved using an enzyme-linked immune assay (ELISA) [20], electrochemical immunoassays [21], liquid chromatography, mass spectrometry, colorimetry [22], fluorescence [23], chemiluminescence [24], and surface-enhanced Raman scattering [25]. Despite their high sensitivity and specificity, these methods require large samples and laborious, complex operations. They also have low stability and high costs [26]. Further, they also often fail to detect the trace amounts of AFP present in these samples. Highly sensitive and specific methods for the quantitative detection of AFP—allowing early stage diagnosis, treatment, and monitoring—are crucial. Therefore, rapid, non-invasive detection of AFP can aid in early stage diagnosis, assist in timely treatment, and reduce the risk of HCC-associated mortality. 

In this review, an overview of biosensing technologies is presented, along with a summary of recent progress in biosensors for sensitive and selective measurement of AFP in biological samples. Representative research on optical, electrochemical, and quartz crystal microbalance-based biosensor strategies is described. Finally, challenges and further prospects for developing ultrasensitive biosensors for AFP detection and early diagnosis of hepatocellular carcinoma are discussed.

## 2. Overview of Biosensing Technology

Biosensors refer to analytical approaches to rapidly measure biospecific interactions through optical, electrochemical, mass, or other signals proportional to the analyte concentration in the reaction mixture [27,28,29]. Self-sufficient, integrated biosensing devices can provide a quantitative or semiquantitative analysis by synergizing a specific biorecognition element with a physio-chemical transducer upon direct contact. There are three major segments: the bioreceptor (or recognition element), the transducer, and the electrical circuit for signal amplification. The recognition element is based on a biological component that interacts with a target analyte, which produces some biological change, resulting in a signal. The transducer and electrical circuit amplify the signal before processing and displaying it as output. Biorecognition elements include enzymes, antibodies, proteins, aptamers, nucleic acids, metabolites, or whole cells. Biosensors are usually classified based on transducer types into (a) electrochemical, (b) optical, and (c) mass-based piezoelectric platforms [30]. Ideal biosensors are affordable, sensitive, specific, user-friendly, rapid, robust, require no added equipment, and are deliverable to the user [31]. The application of biosensors in the biomedical and bioanalytical fields, especially for biomarker detection and early diagnosis, has increased dramatically over the past decade [32,33,34,35]. Researchers have focused mainly on enhancing the limit of detection (LOD), time-dependent sensitivity, and selectivity of the target analyte. So, efforts are directed at improving the affinity of recognition surfaces to biomarkers, using advanced fabrication strategies, modifying surface chemicals, and incorporating nanomaterials [36,37,38,39].

## 3. Optical Methods

Optical-based biosensors are widely used to detect a plethora of cancer biomarkers. Using colorimetry, fluorimetry, Foster Resonance Energy Transfer (FRET), Surface-enhanced Raman Spectroscopy (SERS), Surface Plasma Resonance, and digital holography—among other methods—these sensors can detect trace levels of AFP in samples [40,41,42].

### 3.1. Colorimetric Sensing Substrates for Alpha-Fetoprotein Detection

Several research groups are vigorously exploring the development and application of colorimetric, principle-based biosensing strategies for the early detection of AFP and diagnosis of HCC. In one study, Cao et al. made significant advancements in the application of graphene oxide (GO) for the diagnosis of HCC [43]. Their study revealed a novel and remarkable phenomenon: the dynamic modulation of horseradish peroxidase (HRP) enzymatic activity through exposure to visible light. This allows for precise temporal control over HRP activity, removing the need for agents such as peroxides (typically H_2_O_2_) and acidic stop solutions. The underlying intricacies of this process hinge upon the generation of superoxide anions (O_2_^•−^) and photogenerated holes (h+) when GO is subjected to visible light. These reactive species assume a central role in activating HRP and the subsequent oxidation of standard substrates, notably 3,3′,5,5′-tetramethylbenzidine (TMB) and 2,2′-azino-bis(3-ethylbenzothiazoline-6-sulfonic acid) (ABTS). Moreover, research presented by Cao et al. exemplifies the practical utility of the photo-switchable HRP-GO combination as an efficacious signal reporter in bioassays. One such application is a sandwich immunoassay of AFP, revealing the method’s capacity for sensitivity and selectivity within a linear concentration range spanning from 0.2 fg/mL to 1.0 ng/mL and an LOD of 0.1 fg/mL [43]. Similarly, Zhu et al. developed ZnS nanospheres modified with CdTe quantum dots (QDs). This resulted in a material that exhibited pronounced fluorescence due to the adequate enrichment of the CdTe quantum dots within the porous ZnS nanosphere [44]. The experimental procedure involved the introduction of a reagent known as 2-(5-nitro-2-pyridylazo)-5-(N-propyl-N-sulfopropylamino) phenol, which, upon interaction with the analyte of interest, triggered the release of a substantial quantity of zinc(II) ions. This release forms a purple-hued complex with an absorption peak of 571 nm. Zhao et al. designed a nanomaterial-integrated electrochemical immunosensor to detect AFP quantitatively. Here, the TiO_2_ nanochannel membrane was asymmetrically decorated with platinum titanium nanomaterial (Pt/TiNM), which was utilized to construct an electrochemical immunosensor. Nitroblue tetrazolium (NBT) was used as a substrate that readily reacts with superoxide radical (^•^O_2_), forming a hydrophobic formazan precipitate. The hydrophobic precipitation causes blocks and diminishes the ionic diffusion flux in the nanochannels. The goat antirabbit IgG (Ab_1_) was immobilized on Pt/TiNM, and the detection antibody (Ab_2_) was immobilized on the MoS_2_ nanosheets. In this sandwich-type immunoassay, MoS_2_ nanosheets act as a light-cutting umbrella, inhibiting the photocatalytic reaction, reducing the formazan precipitation, and allowing ionic transport in the nanochannels. This immunoassay can selectively detect AFP concentrations as low as 2 ng/mL [45]. 

**Figure 1:**
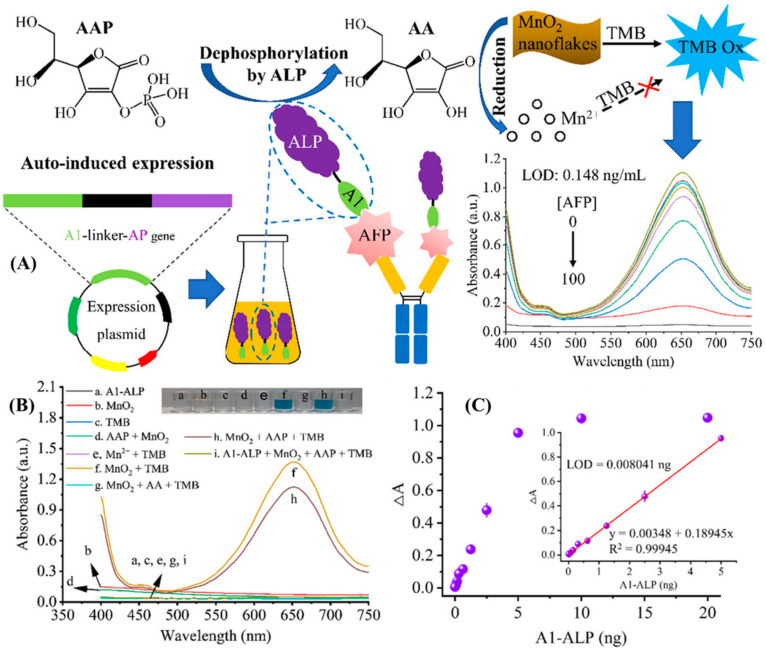
(**A**) Schematic representation of the detection of AFP using enzyme cascade-amplified immunoassay (ECAIA) based on nanobody–alkaline phosphatase fusion (Nb-ALP) and MnO_2_ nanoflakes. (**B**) UV–vis absorption spectra of different combinations of the detection components in the MnO_2_-TMB system. (**C**) Response measurement and correlation analysis in terms of absorbance versus the mass of A1-ALP [22]. Adapted from [22], with copyright permission.

The outcome of this meticulous experimentation is the development of a highly sensitive colorimetric immunoassay. Remarkably, this assay can also serve as a visual diagnostic tool, as it allows for detecting the target biomarker, alpha-fetoprotein (AFP), at remarkably low concentrations. The LOD for AFP in this methodology is an impressive 7 picograms per milliliter (pg/mL). Su et al. pioneered the development of nanomaterials designed to replicate enzymatic functions [22]. This approach yielded a notable advantage over natural enzymes by mitigating the issue of declining catalytic activity. The research team introduced an innovative enzyme cascade-amplified immunoassay (ECAIA), capitalizing on the distinctive properties of nanobodies fused with alkaline phosphatase (Nb-ALP) in conjunction with manganese dioxide (MnO_2_) nanoflakes [22]. Remarkably, the multifunctional biological macromolecule Nb-ALP assumed a dual role, serving as the detection antibody and the reporter molecule. The MnO_2_ nanoflakes were ingeniously employed to mimic the role of oxidase enzymes, facilitating the catalysis of 3,3′,5,5′-tetramethylbenzidine (TMB) into its oxidized blue form. This oxidation process produced a quantifiable signal, detectable at 650 nm (Figure 1). Nb-ALP also demonstrated the capability to dephosphorylate ascorbic acid-2-phosphate (AAP), forming ascorbic acid (AA). This AA, in turn, acted to disintegrate the MnO_2_ nanoflakes, diminishing their oxidation potential and resulting in a decrease in the content of the oxidized TMB. The successful utilization of this TMB-MnO_2_ colorimetric sensing system for Nb-ALP and the optimization of experimental parameters culminated in a highly sensitive detection method. The LOD achieved in this assay was an impressive 0.148 ng/mL [22].

Aydindogan et al. also developed a paper-based colorimetric assay used in the detection of protein biomarkers. Gold nanoparticles (AuNPs) were used for signal production, facilitating the development of a point-of-care (POC) immunoassay. Specifically, relevant antibodies (Abs) were conjugated to AuNPs and subsequently prepared on a nitrocellulose (NC) membrane. Cysteamine-conjugated AuNPs (AuNP-Cys) were anchored onto the NC membrane, and antibodies were affixed to these nanoparticles on the detection pad to establish a spatially organized POC immunoassay. This configuration allowed for testing samples containing two prominent protein biomarkers, alpha-fetoprotein (AFP) and MUC16, which were recognized for their relevance to liver and ovarian cancer [46]. The detection of these tumor markers was made feasible by leveraging changes in the colorimetric properties of AuNPs. In conjunction with color analysis software, a smartphone was employed at the final stage to capture and interpret the visual alterations. The LOD achieved in this study was 1.054 ng/mL for AFP and 0.413 ng/mL for MUC16.

In a prior study by Zhou et al., a dual immunoassay was employed to detect AFP [47]. The signal output system used gold nanoflowers (AuNFs) loaded with fluorescein molecules (AuNF@Fluorescein). To achieve this, AuNFs were modified with thiolated carboxyl ligands, comprising a hydrophobic alkane chain that served as a hydrophobic reservoir for encapsulating fluorescein, a tetra(ethylene glycol) unit for biocompatibility and solubility, and a functional carboxyl group to conjugate biorecognition molecules. Remarkably, the resultant AuNFs had a high loading capacity, accommodating approximately 3.74 million fluorescein molecules per NF, due to their distinctive flower-like structure with numerous intricate branches. At a pH of 8.0, a significant release of fluorescein molecules from AuNF@Fluorescein was observed, leading to amplified fluorescence.

Additionally, fluorescein demonstrated intrinsic peroxidase-like activity, catalyzing the oxidation of TMB when exposed to hydrogen peroxide, thus enabling colorimetric signal generation. The LODs for the fluorescence-based and colorimetric assays were 29 fg/mL and 17.7 pg/mL, respectively. In another study, Zhang et al. developed a composite where Catalytic hemin transformed with biomineralized gold, leading to the formation of a Hemin-Au core [48]. This Hemin-Au core was then encapsulated within a Tb metal–organic framework (MOF) matrix. The catalytic composite materials were utilized to label antibodies in sandwiched immunoassays designed to detect alpha-fetoprotein (AFP). The study’s findings demonstrate the feasibility of conducting immunoassays using Hemin-Au@MOF-based systems, employing two distinct signal amplification mechanisms: hemin-catalyzed chromogenic reaction and gold-catalyzed silver staining. With this approach, the LODs for AFP in the immunoassays were notably low, at 0.020 ng/mL, 0.10 ng/mL, respectively.

### 3.2. Fluorescence Sensing Probes for Alpha-Fetoprotein Detection

**Figure 2:**
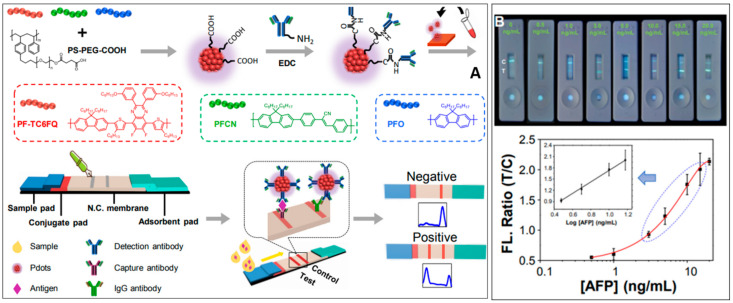
(**A**) Schematic representation of lateral flow assay-based detection of AFP using semiconducting polymer PF-TC6FQ/PFCN/PFO and carboxyl-functionalized polystyrene PS-PEG-COOH. The polymer dots were conjugated with AFP antibody and then loaded onto the conjugate pad. (**B**) Images of the test strips under 365 nm light irradiation after reaction with samples containing AFP antigens (0–20 ng/mL). The bottom panels show their corresponding fluorescence ratios of T/C measured by ImageJ software. The insets in each panel show the calibration curves at antigen concentrations of 3–15 ng/mL. Error bars show standard deviations of 5 replicate measurements [50]. Adapted from [50] with copyright permission.

Micro- and nanomaterials are also actively used to develop fluorescent sensor probes for the ultra-sensitive detection of AFP. A fluorescent immunoassay for AFP was developed using nano graphite carbon nitride (g-C_3_N_4_) as the fluorophore and immunomagnetic beads (MBs) as the separation material [49]. The g-C_3_N_4_, synthesized through thermal polymerization of melamine, carboxylated, and then exfoliated to yield carboxylated nano g-C_3_N_4_ (c-n-g-C_3_N_4_). c-n-g-C_3_N_4_ exhibited excellent fluorescent properties upon characterization. Two antibodies specific to AFP (Ab_1_, Ab_2_) were each conjugated to the MBs and the c-n-g-C_3_N_4_, respectively. The magnetic component, MBs-Ab_1_, forms a sandwich-type complex with the signal component, c-n-g-C_3_N_4_g-C_3_N_4_-Ab_2_, upon AFP addition. The MBs allow for a simpler separation process. The method achieved a noteworthy LOD of 0.43 ng/mL, demonstrating its potential as a fluorescent immunoassay for precise AFP detection. Semiconducting polymer dots (Pdots) have recently gained prominence as novel and biocompatible fluorescent probes due to their remarkable brightness. They are ideally suited for applications in biological and clinical settings. Fang et al. innovatively harnessed Pdots to produce an Immunochromatographic Test Strip (ICTS) for rapid, quantitative screening of prostate-specific antigen (PSA), α-fetoprotein (AFP), and carcinoembryonic antigen (CEA) within a mere 10 min. The construction of the paper-based lateral flow assay, the working mechanism, detection, and analysis are illustrated in Figure 2. The developed Pdot-based ICTS offers swift diagnosis in POC applications. Remarkably, the LOD for AFP in this approach is an impressively low 3.30 pg/mL. This cutting-edge method exhibits promise for the rapid and efficient diagnosis of biomarkers in a clinical setting [50]. Sun et al. developed a nanosensor that utilizes vinyl-functionalized carbon dots (V-CDs) as a transducer and support materials. The AFP nanosensor incorporated N-isopropyl acrylamide (NIPAM) and 4-vinylphenylboronic acid (VPBA) as the responsive monomers to temperature and pH, respectively [51]. The polymerization process was initiated by ammonium peroxodisulphate (APS) and cross-linked using N, N′-methylene bisacrylamide (MBA).

The newly synthesized nanosensor underwent thorough characterization using techniques such as FT-IR, TEM, XRD, and elemental analysis, conclusively confirming successful formation. The fluorescence quenching exhibited by CDs@MIPs showed a favorable linear response to AFP within a range of 10 to 100 ng/mL. The LOD for AFP in this nanosensor system was 0.474 ng/mL. 

A highly sensitive and selective polymeric nanocavity system for the detection of glycoproteins was developed by Morishige et al., employing an innovative molecular imprinting technique known as post-imprinting modification (PIM) [52]. This approach introduced two distinct interaction sites and a fluorescent reporter molecule within each nanocavity. In the PIM-based molecular imprinting process, AFP was modified with polymerizable groups through disulfide bonding, facilitating its immobilization on a substrate functionalized with 4-carboxy-3-fluorophenylboronic acid in a well-organized manner, allowing for the creation of AFP-imprinted nanocavities. Then, a controlled/living radical polymerization, followed by cleavage of disulfide bonds and cyclic diesters, generated AFP-imprinted nanocavities with free thiol groups exclusively located inside these cavities. A thiol-reactive fluorescent dye was introduced into the nanocavities through in-cavity PIM to complete the process. These nanocavities could transduce AFP-binding into changes in fluorescence. The LOD for AFP in this system was 0.27 ng/mL [53].

### 3.3. Luminescence and Fluorescence Resonance Energy Transfer-Based Detection of Alpha-Fetoprotein 

**Figure 3:**
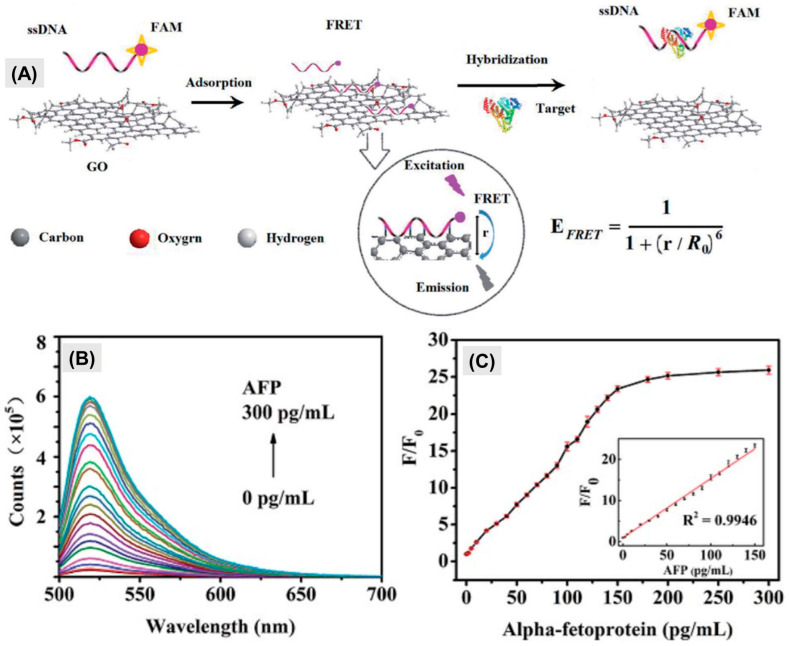
(**A**) Schematic illustration of AFP detection using Graphene Oxide-FAM-AFP-aptamer quenching Mechanism. (**B**) Change in the fluorescence spectra of FAM-ssDNA/GO complex in the presence of increasing concentrations of AFP (0–300 pg/mL). (**C**) The calibration plot of F/F0 of FAM-ssDNA/GO as a function of AFP concentration. Inset: calibration plot of AFP detection (1–150 pg/mL), where F0 and F are the fluorescence signals in the absence and the presence of AFP, respectively. The excitation and emission wavelengths were 488 nm and 520 nm [56]. Adapted from [56], with copyright permission under the terms of the CC-BY-NC-ND 3.0 license.

Fang et al. synthesized two pivotal components tailored for electrochemiluminescence (ECL) applications for luminescence detection of AFP. These components included gold nanoparticles-functionalized graphitic carbon nitride nanosheets (g-C_3_N_4_@AuNPs), designed to serve as the ECL donor, and a palladium nanoparticle-coated metal–organic framework (Pd NPs@NH_2_-MIL-53), engineered to function as the ECL acceptor [54]. g-C_3_N_4_@AuNPs were able to produce robust cathode ECL emission when K_2_S_2_O_8_ was employed as a co-reactant. The AuNPs played a dual role, acting both as accelerators, significantly amplifying and stabilizing the ECL signal, and as connectors for the attachment of Aβ antibodies, thereby augmenting the overall functionality. Concurrently, NH_2_-MIL-53(Al) was used as a label, as it provides structural support to PdNPs. This strategic choice effectively synergized the intensification and broadening of the UV-visible absorption range. Similarly, for the fluorescence resonance energy transfer-based detection of alpha-fetoprotein detection, Zhou et al. produced a straightforward and highly sensitive homogeneous aptasensor for AFP [55]. This aptasensor relies on FRET, utilizing luminescent CdTe quantum dots (QDs) labeled with the AFP aptamer as the donor and gold nanoparticles (AuNPs) functionalized with anti-AFP antibodies as the acceptor. In the presence of AFP, the bio-affinity interactions among the aptamer, target, and antibody bring the QDs and AuNPs into proximity, leading to fluorescence quenching of CdTe QDs through FRET. The LOD was found to be 400 pg/mL, with a linear detection range of 0.5 to 45 ng/mL. The findings of this study are comparable to the work conducted by Wang et al. [56]. Wang and his team developed a novel biosensing platform by integrating a new single-stranded DNA (ssDNA) aptamer and graphene oxide (GO) for highly sensitive and selective detection of AFP [57]. The fundamental principle of this biosensing platform relies on the effective quenching of fluorescence from dye-modified ssDNA upon forming a hybrid structure with graphene oxide–ssDNA (GO-ssDNA), Figure 3. Selective interaction between AFP and GO-ssDNA causes the decomposition of this hybrid structure, leading to fluorescence recovery. The achieved LOD is 0.909 pg/mL, surpassing the sensitivity accomplished by Zhou et al., with a linear detection range of 1 to 150 pg/mL.

Mintz et al. also used carbon dots (C-dots) and gold nanoparticles (AuNPs). Qin et al. developed a highly sensitive aptasensor for detecting AFP [58]. This aptasensor was designed based on fluorescence resonance energy transfer (FRET) between 5-carboxyfluorescein (FAM) and gold nanoclusters (AuNCs). The interactions between AuNCs and FAM-AFP aptamer quenched the fluorescence. Upon the introduction of AFP into the FAM-AFP aptamer-AuNCs FRET system, the AFP selectively bound to its aptamer, inducing a conformational change and significantly reducing the coordination interaction between the AFP aptamer and AuNCs leading to a recovery of fluorescence. The LOD for AFP in this system is 6.631 ng/mL, and the linear range of detection spans from 10.0 to 100.0 ng/mL.

### 3.4. Surface-Enhanced Raman Spectroscopy and SPR-Based Detection of Alpha-Fetoprotein 

**Figure 4:**
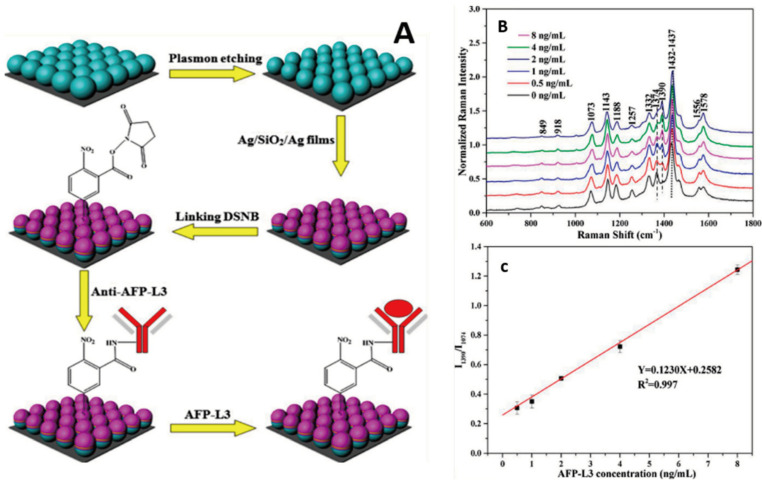
(**A**) Illustration of PS@Ag/SiO_2_/Ag chips fabrication process and chemisorption of the DSNB molecules on the SERS substrates, with subsequent covalent binding of anti-AFP-L3 and followed by capture of the corresponding antigen AFP-L3. (**B**) SERS spectra of DSNB absorbed on the Ag/SiO_2_/Ag chips conjugated with antibody and anti-AFP-L3 capture with the AFP-L3 antigen of different concentrations (0, 0.5, 1, 2, 4, and 8 ng/mL). (**C**) The calibration plot of the intensity ratio of I_1390_/I_1074_ against the AFP-L3 concentration [60]. Adapted from [60] with copyright permission.

To detect AFP using Surface-enhanced Raman spectroscopy, Zhu et al. designed a SERS-active substrate by creating nanocone arrays through nanosphere lithography [59]. They then fine-tuned to reduce the distance between adjacent Au nanocones and modify the sharpness of the tips, enhancing the SERS signals. The sensor achieved an LOD of 0.5 ng/mL for AFP-L3. Two years before Zhu et al.’s research, Zhang et al. also developed a novel SERS-active chip for detecting AFP-L3 [60]. This innovative chip utilized a two-dimensional, non-close-packed polystyrene colloid sphere array composite structure with different sizes as the SERS-active substrate. The SERS probe consisted of an antibody-conjugated Ag/SiO_2_/Ag surface labeled with 5,5-Dithiobis (succinimidyl-2-nitrobenzoate) (DSNB), as shown in Figure 4. The study demonstrated a robust linear relationship between the concentration of AFP-L3 and the Raman intensity ratio, achieving an LOD at 0.078 ng/mL and a linear detection range of 0–8 ng/mL.

Similarly, Wang et al. developed a novel SERS biosensing platform that integrates a target-responsive DNA hydrogel for the sensitive detection of AFP [25]. The DNA hydrogel’s linker strand, functioning as an aptamer, specifically recognizes AFP and controls the release of immunoglobulin G (IgG) encapsulated within the hydrogel. Upon encountering AFP, the hydrogels disentangle, facilitating IgG release. Subsequently, the liberated IgG is captured by SERS probes and biofunctional magnetic beads, forming sandwich-like structures. This process leads to a decrease in the signal of Raman tags in the supernatant after magnetic separation. This platform’s LOD is 50 pg/mL, with a linear detection range from 50 pg/mL to 0.5 μg/mL. Gao et al. devised a Surface-Enhanced Raman Scattering (SERS)-based immunoassay, leveraging Au-Ag alloy nanoparticles and Ag/AgBr hybrid nanostructures to detect AFP [61]. The Au-Ag alloy nanoparticles, serving as typical bimetal or metal/semiconductor plasmonic materials, exhibit strong SERS enhancement characteristics and excellent monodispersity. Simultaneously, the Ag/AgBr hybrid nanostructure demonstrates remarkable stability. This approach achieves an LOD of 1.86 fg/mL, marking the lowest LOD among all the SERS methods discussed in this review. The linear detection range spans from 2 fg/mL to 0.8 μg/mL [61].

To detect alpha-fetoprotein using the surface-plasmon resonance phenomenon, Wang et al. developed an SPR-based sensing system using antibody-QD conjugates for ultrasensitive detection of multiple tumor markers, including AFP, in clinical specimens. The authors employed a dual signal-amplification technique. The surface modification with AuNP-antibody and antibody-QD conjugates significantly enhanced the signal response (50-fold) with a LOD as low as 0.1 ng/mL for AFP. The authors demonstrated that results obtained using the proposed SPR biosensor were consistent with electrochemiluminescence-based detection system [62].

## 4. Electrochemical Sensor-Based Detection of Alpha-Fetoprotein

Electrochemical sensors usually detect specific chemical compounds or analytes in a sample. Based on their working principles, electrochemical sensors measure the binding interactions in a reaction mixture and generate highly sensitive and selective electrical signals as output readouts. These sensors widely detect targets, including bacterial or viral pathogens and biomarkers necessary for diagnosing various diseases. These versatile sensors include genosensors, protein sensors, immunosensors, aptasensors, and whole-cell sensors. Several electrochemical sensing strategies have been reported for the detection of AFP. 

### 4.1. Electrochemical Aptasensor-Based Detection of Alpha-Fetoprotein 

**Figure 5:**
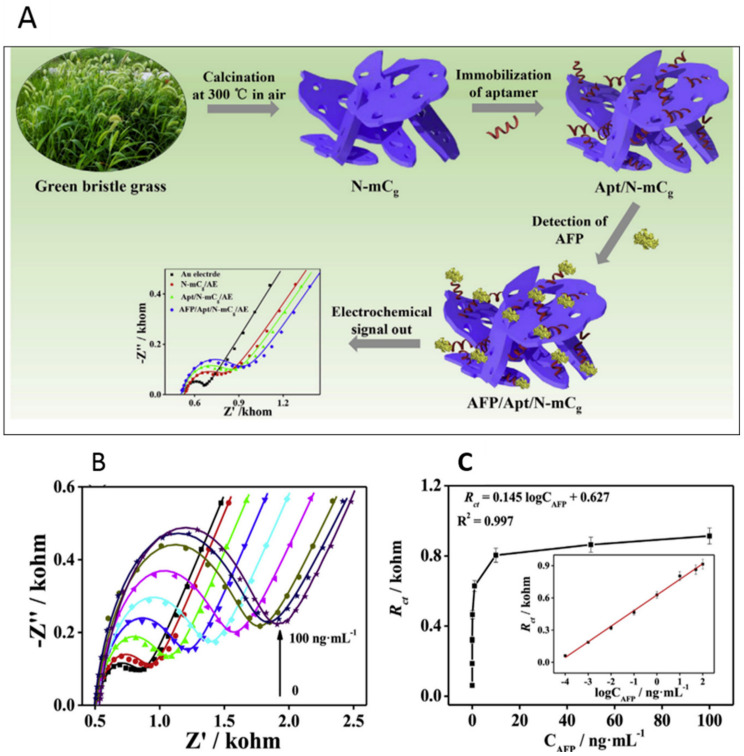
(**A**) Schematic representation of nitrogen-doped nanoporous carbon nanomaterials (N-mC)-based aptasensor for the detection of AFP. (**B**) Electrochemical impedance spectroscopy (EIS) Nyquist plots for the detection of different concentrations of AFP (0.0001, 0.001, 0.01, 0.1, 1, 10, 50, and 100 ng/mL) using the N-mCg-based aptasensor; (**C**) The corresponding calibration curves between DRct and AFP concentrations (inset: the linear fit plot of DRct as a function of the logarithm of AFP concentration (n = 3) [66]. Adapted from [66], with copyright permission.

Yang et al. developed an electrochemical aptasensor for detecting AFP utilizing a graphene oxide (GO)-based platform [63]. This aptasensor was constructed by covalently immobilizing an AFP-specific aptamer with NH_2_ functionality onto the carboxyl-rich GO. The practicality of the fabrication procedures was thoroughly investigated using cyclic voltammetry (CV) and electrochemical impedance spectra (EIS) analysis. CV was employed to detect the signal changes of the aptasensor, revealing an LOD of 3 pg/mL and a linear range of detection spanning from 0.01 to 100 ng/mL. Four years later, Rahmati et al. engineered an aptasensor for AFP detection, utilizing the hollow N-C n-box skeletons derived from Fe_2_O_3_ nanocubes as the template [64]. Developing a label-free electrochemical aptasensor involved the covalent immobilization of NH_2_-functionalized aptamer on Ni(OH)_2_@N-C n-box, serving as an efficient substrate. Electrochemical evaluations conclusively demonstrated a linear relationship between AFP concentrations and charge transfer resistance (Rct), achieving an LOD of 0.3 fg/mL. This LOD is significantly lower than the threshold established by the work conducted by Yang et al. The aptasensor also exhibited a broad linear detection range from 1 fg/mL to 100 ng/mL. Yang et al. pioneered the development of an electrochemical aptasensor featuring an incredibly low LOD [65]. This novel bi-directionally amplified ratiometric electrochemical aptasensor utilized exonuclease-assisted target recycling and a bioconjugate probe for the ultrasensitive detection of AFP. The aptasensor design involved attaching a ferrocene-labeled capture probe (Fc-CP) onto the electrode surface through hybridization with thiolated DNA1. The sensor achieved an ultralow LOD of 269.4 ag/mL, highlighting its exceptional sensitivity. The linear detection range spanned seven orders of magnitude. Huang et al. also introduced a remarkable nitrogen-doped nanoporous carbon nanomaterial specifically derived from grass (N-mCg). Among the various carbon derivatives, N-mCg stood out with the lowest degree of carbon defect, the highest ID/IG ratio in Raman spectra, and an impressive specific surface area of 186.2 m^2^/g [66]. These characteristics translate into excellent electrochemical activity and a strong affinity toward aptamer strands (Figure 5). Employing the EIS method, the sensor achieved a low LOD at 60.8 fg/mL for AFP, with a linear range spanning from 0.1 pg/mL to 100 ng/mL. This underscores the high sensitivity and broad detection range of the aptasensor for AFP quantification.

Similarly, Wei et al. developed an innovative sensor using two distinct electrochemical signals generated by synthesized nanoparticles [67]. The sensor incorporated 4-mercaptophenylboronic acid (MPA)-functionalized copper nanoparticles (MPA-CuNPs) and Lens culinaris agglutinin (LCA)-functionalized silver nanoparticles (LCA-AgNPs). This design enabled the simultaneous quantification of Lens culinaris agglutinin-reactive Alpha-fetoprotein (AFP-L3) and total AFP in serum samples, facilitating the direct electrochemical assay of AFP-L3%. The sensor demonstrated an LOD of 0.01 ng/mL and exhibited a linear detection range of 0.4 to 1000 ng/mL. In another study, Zhai et al. engineered an alpha-fetoprotein (AFP) sensor utilizing a gold microelectrode denoted as AuμE [68]. The sensor employed square wave voltammetry with [Fe(CN)_6_]^3−/4−^ as a mediator. The assay incorporated a sulfhydryl-modified AFP aptamer as a biorecognition element, directly conjugated onto the AuμE surface for precise AFP capture. This sensor demonstrated an LOD of 25 pg/mL and linear detection from 100 pg/mL to 100 ng/mL.

Upan et al. engineered an electrochemical sensor using platinum nanoparticles on carboxylated-graphene oxide (PtNPs/GO-COOH), used to modify a screen-printed graphene-carbon paste electrode (SPGE) as the immobilization platform, where the bio-recognition element employed was an AFP aptamer [69]. Incorporating synthesized GO-COOH increased the surface area and quantity of immobilized aptamer. Following this, PtNPs were used on GO-COOH to augment electrical conductivity and enhance the oxidation current of the hydroquinone electrochemical probe. The aptamer exhibited selective interaction with AFP, resulting in an LOD of 1.22 ng/mL and linear detection from 3.0 to 30 ng/mL.

### 4.2. Electrochemical Immunosensor-Based Detection of Alpha-Fetoprotein 

To apply an immunoassay-based sensing technique, Li et al. innovatively developed potentiometric immunosensors for the sensitive detection of alpha-fetoprotein (AFP) in hepatocellular carcinoma using distinct electrodes, including a carbon fiber microelectrode (CFME) and carbon-disk electrode (CDE) [70]. The immunosensor construction involved the initial covalent conjugation of anti-AFP capture antibodies onto activated electrodes through typical carbodiimide coupling. Subsequently, a one-step immunoreaction protocol was successfully implemented, establishing a novel potentiometric immunoassay upon the introduction of AFP. This immunosensor exhibits an LOD of 3.2 pg/mL, with a linear detection range of 0.1 to 100 ng/mL. In the subsequent year, Chen et al. introduced an immunosensor with a notably lower LOD for the sensitive detection of AFP [71]. This innovative research presents a sandwich-type electrochemical immunosensor designed for the ultrasensitive detection of AFP using a sensing platform based on spherical nucleic acids-templated silver nanoclusters (AgNCs). In this design, DNA-functionalized gold nanoparticles (AuNPs@DNA) act as both the crosslinker to immobilize the primary antibody (anti-AFP antibody 1, Ab_1_) and as the template for synthesizing AgNCs on AuNPs to form AuNPs@DNA-AgNCs. Simultaneously, p-sulfonated calyx[4]arene (pSC4)-modified Au was employed to immobilize the secondary antibody (anti-AFP antibody 2, Ab_2_) through host–guest recognition between Ab_2_ and pSC4. Upon encountering AFP, the immunoreaction signal undergoes significant amplification through the electrochemical reduction of AgNCs, resulting in an LOD of 7.74 fg/mL and a linear detection range spanning from 0.001 to 100 ng/mL. Jiang et al. also developed an innovative electrochemical immunosensor utilizing polydopamine-coated Fe_3_O_4_ nanoparticles (PDA@Fe_3_O_4_ NPs) as a smart label, employing polyaniline (PANI) and AuNPs as substrate materials for AFP detection [72]. It showed an LOD of 254 pg/mL, with a linear detection range of 1 pg/mL to 100 ng/mL. Rong et al. developed an electrochemical immunosensor for the detection of AFP by leveraging the unique properties of ordered mesoporous carbon (OMC)@AuNPs composites and AuPt-methylene blue (AuPt-MB) [73]. This sensor, designed as a disposable ultrasensitive sandwich, utilized AuNPs to immobilize AFP-Ab_1_ through Au-N bonds. The rod-like AuPt-MB, a novel redox-active species, exhibited high, uniform morphology and excellent biocompatibility. These features enabled it to both anchor AFP-Ab_2_ and release electrochemical signals. The immunosensor achieved an LOD of 3.33 fg/mL and offered linear detection from 10 fg/mL to 100 ng/mL. 

Similarly, Li et al. devised an immunosensor for the detection of AFP. Initially, they prepared the AFP heptamer fusion protein (A1-C4bp α) by fusing the AFP-specific nanobody (A1) with the C-terminal fragment of C4-binding protein (C4bp α) [74]. The A1-C4bp α heptamer, characterized by heightened affinity, was employed as the recognizing probe to efficiently capture numerous AFP molecules, amplifying the sensitivity of the immunosensor. Additionally, the AuNPs-decorated zeolitic imidazolate framework (AuNPs@ZIF-8), known for its extensive surface area and abundant binding sites, acted as a nanocarrier to enhance heptamer loading. Incorporating multi-walled carbon nanotubes (MWCNTs) with excellent electrical conductivity onto a glassy carbon electrode (GCE) further contributed to augmenting electrochemical signals. The immunosensor demonstrated an LOD of 0.033 pg/mL with linear detection from 0.1 to 105 pg/mL. Li et al. developed a label-free electrochemical immunosensor by preparing worm-like platinum (WL Pt) nanomaterial through chemical etching without organic solvents or ultra-high temperatures. The WL Pt nanomaterial, characterized by its small particle size and the formation of surface vacancies during etching, enhanced electrocatalytic activity for H_2_O_2_ reduction due to its improved surface area, which was used for signal amplification. With an antibody-based sandwich-like strategy for antigen recognition, this sensor demonstrated an LOD of 0.028 pg/mL and a linear detection range spanning from 0.0001 to 100 ng/mL for AFP detection [75]. Feng et al. devised a stable and sensitive label-free electrochemical immunosensor utilizing flower-like gold microstructures/polyaniline/reduced graphene oxide/Prussian blue (HFG/PANI/rGO/PB) composite-modified electrode [76]. This immunosensor was explicitly designed to determine AFP and showed an LOD of 0.003 ng/mL and linear detection of 0.01–30 ng/mL. Shi et al. developed an immunosensor using a glassy carbon electrode (GCE) whose substructure and geometry were initially modeled through first-principles density functional theory (DFT) by employing AFPD/3−21 G. Isoorientin served as a model to investigate the effect of adsorbate bonding on the GCE surface by both experimental and computational methods, confirming that the modification of isoorientin onto GCE occurred through non-covalent interactions. Subsequently, the Isoorientin/Anti-alpha-fetoprotein (AFP) antibody-modified GCE was prepared by sequentially depositing isoorientin and anti-AFP antibody (Ab) onto the GCE surface, creating an immunosensor for the detection of AFP with an LOD of 0.2 pg/mL and a linear detection range of 0.001–10 ng/mL [77].

Zhao et al. developed a sensor for detecting AFP utilizing a Cu_2_O spherical nanoparticle reduced by hydrothermal reaction to create a label-free electrochemical immunosensor with an innovative signal-amplification strategy [78]. Cuprous oxide was integrated with multi-walled carbon nanotubes, and then Au NPs were adsorbed to enhance both the electrochemical signal and attachment of AFP antibodies. The sensor exhibited a low LOD of 0.33 pg/mL and a linear detection range of 0.001 to 40 ng/mL. 

## 5. Mass-Based Sensor-Based Detection of Alpha-Fetoprotein 

Mass-based sensors are emerging as handy tools used for biosensing due to their high sensitivity and specificity. They operate on the principle that a mass change is observed when the target biomolecule binds to the sensor’s surface. These mass-based sensors are of two types: cantilever and piezoelectric (piezo) sensors, which are used in diagnosing HCC. 

### 5.1. Mass-Based Piezoelectric Sensor-Based Detection of Alpha-Fetoprotein 

Su et al. developed a mass piezo sensor to detect AFP [79]. The sensor utilized a commercially available lead zirconate titanate (PZT) ceramic resonator with a high resonance frequency as the transducer for a piezoelectric biosensor. The design incorporated a dual ceramic resonator scheme, connecting two ceramic resonators—one for sensing and one for control—in parallel. This configuration effectively minimized environmental influences, including temperature fluctuations, and ensured the necessary frequency stability for biosensing. The sensor’s LOD was determined to be 0.25 ng/mL, and the linear range of detection spanned from 2.5 ng/mL to 2.5 × 10^2^ ng/mL.

### 5.2. Mass-Based Cantilever Sensor-Based Detection of Alpha-Fetoprotein 

Li et al. developed a mass-based sensor for detecting AFP utilizing antibodies [80]. Their approach involved a microcantilever array biosensor based on a sandwich structure, allowing simultaneous measurement of carcinoembryonic antigen (CEA) and AFP. An optical readout technique involved real-time monitoring of the cantilever profile. Initially, aptamers specific to CEA and AFP were self-assembled on their respective cantilevers. After adsorbing CEA and AFP, antibodies for each target were added. The formation of the aptamer–antigen–antibody sandwich structure on the gold surface induced compressive stress on the cantilever, bending it. The real-time profile of the cantilever could be monitored. The sensor demonstrated a LOD of 0.6 ng/mL and a linear range of detection spanning from 1 to 900 ng/mL. Zhao et al. developed a sensor for detecting AFP utilizing a microcantilever biosensor with AuNPs amplification [81]. This innovative approach enhances sensitivity to AFP. The biosensor achieved a remarkable LOD of 21 pg/mL and a linear range of detection spanning from 10–70 ng/mL, providing a broad dynamic range for accurate and reliable measurements across a range of concentrations (Table 1).

## 6. Conclusions and Future Perspectives

In this review, many recent methodologies were discussed for the sensitive detection of AFP in buffers, as proof of concepts, and in biological samples. Many analytical techniques, such as biofunctionalization, sensor fabrication using nanomaterials, surface modifications, self-assembly, and other modern methods, have been implemented to design and construct AFP biosensors. These biosensors perform very well and can detect AFP in a wide range of concentrations. They have significant stability and are highly reproducible, specific, and sensitive. Most of the developed biosensors are used for the direct application of AFP from serum samples, and this is a promising alternative to the conventional methods for diagnosis. There is a wide diversity of biosensors for detecting AFP, suggesting the high future demand for these devices. Moreover, these miniaturized biosensing devices are low-cost, more accurate, and user-friendly; along with their applicability, they can be easily applied to point-of-care applications in areas with limited access to clinical facilities. Despite biosensors having many advantages, they have limitations as well. For example, labeling may influence an optical sensor. The accuracy of real sample analysis using the fluorescence method may be affected by co-existing biomolecules, solid and semisolids, or colloidal particles due to autofluorescence and light scattering. Colorimetric biosensors for detecting AFP are more advantageous due to visual detection by the naked eye. An ultrasensitive detection of AFP has been achieved by photoswitching the enzymatic activity of horseradish peroxidase using GO as a colorimetric immunoassay sensing platform. This results in the LOD of 0.1 fg/mL [43]. On the other hand, the colorimetric assay suffers from needing enzymes [82]. Another limitation of the colorimetric assay occurs when using salt-induced AuNps, where the biological application of this method suffers from AuNps aggregation by the biological matrix in the sample [83]. The combination of electrochemical and luminescence methods (ECL) certainly has potential and is sensitive to AFP diagnosis; however, the sensitivity of this method highly depends on the redox molecules used. The photoelectrochemical sensing technique has its unique advantage due to integrating fluorescence and electrochemical methods. Although this method is sensitive, separating excitation sources and photocurrent signal detection is challenging, leading to background noise and reducing sensitivity. FRET-based biosensors depend on the distance between the labeled donor and acceptor fluorophores. The fluorophore labeling and the specific interaction of biological molecules may influence the actual distance and affect the accuracy of the sensors. LSPR and SERS methods are very sensitive, but they come with a more complex construction pathway [84]. The integration of fiber optic probes and fluorescence chips with the method mentioned above would significantly enhance the accuracy of AFP detection. Fiber optic-based biosensors may be of more interest for POC testing due to their portable size, high sensitivity, accuracy, easy operation, real-time monitoring, and in vivo diagnosis, as well as no light scattering from the whole blood. Additionally, mineralized label-free electrochemical biosensors are, compared to other optical methods, more reliable and accurate for detecting AFP directly from biological [85]. The instrument-based analysis of biological samples requires well-equipped, highly sophisticated instruments, expensive professional technicians, and a long time to obtain the results. Therefore, the future of HCC could be a rapid analysis with a low-cost, miniaturized biosensing device for POC diagnosis. This would be helpful for patients to self-monitor the progress of the disease by determining it themselves, and it would be more convenient for those who may not have easy access to clinics or hospitals. A microfluidic chip incorporating high-throughput screening may also be an ideal device for POC applications with high accuracy and low cost, allowing mass screening irrespective of economic barriers and geographical locations. 

## Figures and Tables

**Table 1:** Various bioanalytical methods used for the detection of Alpha-Fetoprotein.

S. No	Detection Method	LOD	Range	Advantages	References
1	Colorimetric	0.1 fg/mL	0.2 fg/mL to 1.0 ng/mL	High catalytic ability, precise time control, and free of harmful reagents	[43]
2	Colorimetric	10 pg/mL	0.05 ng/mL to 12 ng/mL	High accuracy due to the dual-detection feature	[44]
3	Colorimetric	2 ng/mL	2 ng/mL to 10 µg/mL	Capable of detecting other immune targets, increasing usability	[45]
4	Colorimetric	1.054 ng/mL	0.1 ng/mL–100 ng/mL	Capable of detecting MUC-16 biomarker used in ovarian cancer	[46]
5	Colorimetric/Fluorescence	29 fg/mL (fluorescence)	10 fg/mL to 10,000 fg/mL (fluorescence)	It can be used for ultrasensitive detection via the fluorescence mode and standard diagnosis via the colorimetric mode	[47]
		17.7 pg/mL (colorimetric)	5 pg/mL to 5000 pg/mL (colorimetric)		
6	Colorimetric	0.020 ng/mL	0.25 to 38 ng/mL	The methodology can be used for the fabrication of other enzymes or enzymatic mimics	[48]
7	Fluorescence	0.43 ng/mL	5 ng/mL to 600 ng/mL	Good sensitivity, excellent selectivity, high biocompatibility, and low cost; the results can be used for further detection of biomarkers in complex matrices	[49]
8	Fluorescence	3.30 pg/mL	3 ng/mL to 15 ng/mL	First polymer dot-based immunochromatography strip capable of multiplex detection	[50]
9	Fluorescence	0.474 ng/mL	10 to 100 ng mL^−1^	This method is rapid, convenient, and highly selective	[51]
10	Fluorescence	0.27 ng/mL	0.5 to 100 ng/mL	This technique can be used for ELISA-relevant, antibody-free sensing of glycoproteins	[52]
11	Luminescence	3.4 fg/mL	10 fg/mL to 50 ng/mL	It can be used as a tool for analysis for trace detection of sensitive molecules in clinical analysis	[54]
12	FRET	400 pg/mL	0.5 ng/mL to 45 ng/mL	This method can be modified and used for the testing various biomarkers in POC analysis	[55]
13	FRET	0.909 pg/mL	1 pg/mL to 150 pg/mL	It can be used for serum and cell imaging for the detection of AFP	[56]
14	FRET	6.631 ng/mL	10 ng/mL to 100 ng/mL	The sensor has a simple configuration and can be modified to detect various biomarkers	[58]
15	SERS	0.5 ng/mL	0.5 to 4 ng/mL	Can detect AFP-L3, which is better suited for early diagnosis of HCC	[59]
16	SERS	0.078 ng/mL	0 ng/mL to 8 ng/mL	Can detect AFP-L3, which is better suited for early diagnosis of HCC	[60]
17	SERS	1.86 fg/mL	2 fg/mL to 0.8 μg/mL	The sensor is ultrasensitive and can be used for HCC point-of-care testing	[61]
18	Aptasensor	3 pg/mL	0.01 ng/mL to 100 ng/mL	It is simple to manufacture, cost-effective, and has a wide linear range of detection	[63]
19	Aptasensor	0.3 fg/mL	1 fg/mL to 100 ng/mL	The detection capabilities were tested with real live samples and proved superior and reliable	[64]
20	Aptasesnsor	269.4 ag/mL	0 fg/mL to 1 µg/mL	It has an ultralow detection limit and a wide range of detection of seven orders of magnitude	[65]
21	Aptasensor	60.8 fg/m	0.1 pg/mL to 100 ng/mL	Has high reproducibility and good stability in serum samples of cancer patients	[66]
22	Aptasensor	0.01 ng/mL	0.4 ng/mL to 1000 ng/mL	Can detect AFP-L3, which is better suited for early diagnosis of HCC	[67]
23	Aptasensor	2.5 × 10^−11^ g/mL	10^−10^ g/mL to 10^−7^ g/mL	Real blood samples were used, and they correlated with the results provided by the electrochemiluminescence assay. This one-step assay is perfect for point-of-care diagnostic	[68]
24	Aptasensor	1.22 ng/mL	3 ng/mL to 30 ng/mL	Has high sensitivity to AFP molecules, good stability, and high recovery rates	[69]
25	Aptasensor	3.2 pg/mL	0.1 ng/mL to 100 ng/mL	It has a high sensitivity, good reproducibility, and the results are consistent with ELISA	[70]
26	Immunosensor	7.74 fg/mL	0.001 ng/mL to 100 ng/mL	It is an ultrasensitive sensor with a broad detection range and has excellent reliability and reproducibility	[71]
27	Immunosensor	0.254 pg/mL	1 pg/mL to 100 ng/mL.	The sensor has high levels of stability, repeatability, and selectivity	[72]
28	Immunosensor	3.33 fg/mL	10 fg/mL to 100 ng/mL	The device demonstrated excellent selectivity, high stability, and outstanding reproducibility	[73]
29	Immunosensor	0.033 pg/mL	0.1 to 10^5^ pg/mL	The nanobody multimerization strategy can be used to enhance sensitivity and detection capability in other methods	[74]
30	Immunosensor	0.028 pg/mL	0.0001 ng/mL to 100 ng/mL	The method makes use of chemical etching rather than ultra-high temperatures, thus making it affordable, and has excellent detection capabilities	[75]
31	Immunosensor	0.003 ng/mL	0.01 ng/mL to 30 ng/mL	It can be used with several other antibodies with minor modification, detects AFP well, and can be used for clinical HCC diagnosis	[76]
32	Immunosensor	0.0002 ng/mL	0.001 ng/mL to 10 ng/mL	It has an ultralow limit of detection and a wide linear range	[78]
33	Immunosensor	0.33 pg/mL	0.001 ng/mL to 40 ng/mL	The sensor has been tested with human serum and has demonstrated good reproducibility and excellent long-term stability	[78]
34	Piezoelectric	0.25 ng/mL	2.5 ng/mL to 2.5 × 10^2^ ng/mL	This sensor can be used with different chemical interfaces and can be developed to detect multiple analytes simultaneously	[79]
35	Microcantilever	0.6 ng/mL	1 ng/mL to 900 ng/mL	This biosensor is capable of detecting two biomarkers simultaneously, thus highlighting its potential in detecting other biomarkers simultaneously for early clinical diagnosis	[80]
36	Microcantilever	21 pg/mL	10 ng/mL to 70 ng/mL	This sensor’s gold nanoparticle amplificant method amplified the detection capabilities about 70 times. This can be further worked on and employed in different sensors to establish a lower limit of detection	[81]
